# Potential of a Novel Chemical Compound Targeting Matrix Metalloprotease-13 for Early Osteoarthritis: An In Vitro Study

**DOI:** 10.3390/ijms23052681

**Published:** 2022-02-28

**Authors:** Junko Inagaki, Airi Nakano, Omer Faruk Hatipoglu, Yuka Ooka, Yurina Tani, Akane Miki, Kentaro Ikemura, Gabriel Opoku, Ryosuke Ando, Shintaro Kodama, Takashi Ohtsuki, Hirosuke Yamaji, Shusei Yamamoto, Eri Katsuyama, Shogo Watanabe, Satoshi Hirohata

**Affiliations:** 1 Department of Cell Chemistry, Okayama University Graduate School of Medicine, Dentistry and Pharmaceutical Sciences, Okayama 700-8558, Japan; jinagaki@cc.okayama-u.ac.jp; 2 Department of Medical Technology, Graduate School of Health Sciences, Okayama University, 2-5-1, Shikata-cho, Okayama 700-8558, Japan; pe5f1h1v@s.okayama-u.ac.jp (A.N.); p6vz88lj@s.okayama-u.ac.jp (Y.O.); prqe6nwh@s.okayama-u.ac.jp (Y.T.); p3uo4rz5@s.okayama-u.ac.jp (A.M.); pn229drz@s.okayama-u.ac.jp (K.I.); gabbyjnr53@gmail.com (G.O.); pc782bqw@s.okayama-u.ac.jp (R.A.); pybv6bh1@s.okayama-u.ac.jp (S.K.); pebm12z8@okayama-u.ac.jp (T.O.); p7f11dre@s.okayama-u.ac.jp (S.Y.); pbi076aa@okayama-u.ac.jp (E.K.); watanabe1224@okayama-u.ac.jp (S.W.); 3 Department of Pharmacology, Faculty of Medicine, Kindai University, Higashi-Sayama, Osaka 577-8502, Japan; farukjp@hotmail.com; 4 Heart Rhythm Center, Okayama Heart Clinic, Takeda 54-1, Okayama 703-8251, Japan; yamaji2@mac.com

**Keywords:** osteoarthritis, matrix metalloproteinase, MMP13, ADAMTS9, expression screening, chondrocytes

## Abstract

Osteoarthritis is a progressive disease characterized by cartilage destruction in the joints. Matrix metalloproteinases (MMPs) and a disintegrin and metalloproteinase with thrombospondin motifs (ADAMTSs) play key roles in osteoarthritis progression. In this study, we screened a chemical compound library to identify new drug candidates that target MMP and ADAMTS using a cytokine-stimulated OUMS-27 chondrosarcoma cells. By screening PCR-based mRNA expression, we selected 2-(8-methoxy-2-methyl-4-oxoquinolin-1(4H)-yl)-N-(3-methoxyphenyl) acetamide as a potential candidate. We found that 2-(8-methoxy-2-methyl-4-oxoquinolin-1(4H)-yl)-N-(3-methoxyphenyl) acetamide attenuated IL-1β-induced MMP13 mRNA expression in a dose-dependent manner, without causing serious cytotoxicity. Signaling pathway analysis revealed that 2-(8-methoxy-2-methyl-4-oxoquinolin-1(4H)-yl)-N-(3-methoxyphenyl) acetamide attenuated ERK- and p-38-phosphorylation as well as JNK phosphorylation. We then examined the additive effect of 2-(8-methoxy-2-methyl-4-oxoquinolin-1(4H)-yl)-N-(3-methoxyphenyl) acetamide in combination with low-dose betamethasone on IL-1β-stimulated cells. Combined treatment with 2-(8-methoxy-2-methyl-4-oxoquinolin-1(4H)-yl)-N-(3-methoxyphenyl) acetamide and betamethasone significantly attenuated MMP13 and ADAMTS9 mRNA expression. In conclusion, we identified a potential compound of interest that may help attenuate matrix-degrading enzymes in the early osteoarthritis-affected joints.

## 1. Introduction

Osteoarthritis (OA) is a chronically progressive joint disease caused by various factors [1,2]. Several mechanical and biological factors including age, obesity, trauma, inflammation, and genetic susceptibility contribute to the development of OA [3,4]. OA is managed using nonsteroidal anti-inflammatory drugs (NSAIDs) and analgesics are the first-line drugs. One strategy for managing OA is to introduce supportive drugs/chemical compounds that can be used in combination with existing OA treatments. One counterplan is to introduce supportive drug/chemical compounds that can be used in combination with existing OA treatments. Articular cartilage is mainly composed of chondrocytes and extracellular matrix (ECM) including collagen and proteoglycans [5,6]. Although the mechanism of OA development remains unclear, disruption of the balance between anabolic and catabolic signaling pathways is known to cause articular ECM cartilage. In OA-affected cartilage, ECM proteins are degraded by the aberrant upregulation of ECM-degrading proteinases such as matrix metalloproteinases (MMPs) and aggrecanases (a disintegrin and metalloproteinase with thrombospondin motifs (ADAMTSs)). MMPs, especially MMP13, and ADAMTS4, ADAMTS5, and ADAMTS9, are key players in OA development [6,7,8,9]. Therefore, these proteinases, represented by MMP13 and ADAMTS4/5, are intriguing target molecules for treating OA. Therefore, the goal of this study was to identify novel supportive molecules that may suppress ECM-degrading proteinases. In this study, we focused on identifying chemical compounds targeting MMPs and ADAMTS. We identified a novel promising candidate with an effect additive to that of betamethasone.

## 2. Results

### 2.1. Screening of Candidate Compounds Based on Cytotoxicity

**Figure 1:**
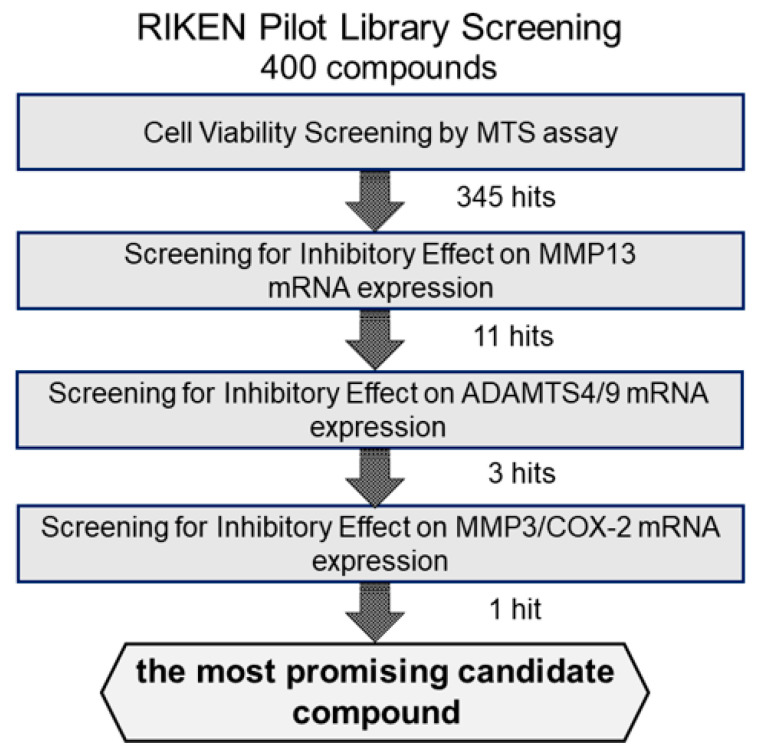
Schematic representation of the strategy for screening the candidate compounds. To evaluate the cytotoxicity of candidate compounds, OUMS27 cells were treated with 10 µg/mL each test compound for 24 h, followed by an MTS assay for cell viability estimation (*n* = 3 or *n* = 5). Among the 400 test compounds we began with, 345 had the least cytotoxic effects compared to the untreated cells (*p* < 0.05). We next screened these 345 compounds for their potential inhibitory effect on IL-1β-induced MMP13 mRNA expression and selected 11 most effective compounds. Subsequently, we assessed the inhibitory effect of these 11 compounds on the IL-1β-induced ADAMTS4 and ADAMTS9 mRNA expression along with MMP3 and COX-2 mRNA expression and finally selected the most promising candidate compound: (2-(8-methoxy-2-methyl-4-oxoquinolin-1-yl)-N-(3-methoxyphenyl)acetamide).

We first examined the cytotoxicity of the 400 compounds in OUMS27 cells using the MTS assay. Cells were treated with 10 μg/mL of each test compound in the library and incubated for 24 h. Cell viability was expressed as the percentage of proliferation compared with the control cells, which were treated with only 1.0% DMSO. The compounds showing cell viabilities of 80% or less were considered as highly (strongly) cytotoxic. Among the 400 compounds, 55 were determined to have strong cytotoxic effects; therefore, the remaining 345 compounds were used in subsequent experiments (Figure 1).

### 2.2. Screening via Inhibitory Effects on IL-1β-Induced MMP13 mRNA Expression

**Figure 2:**
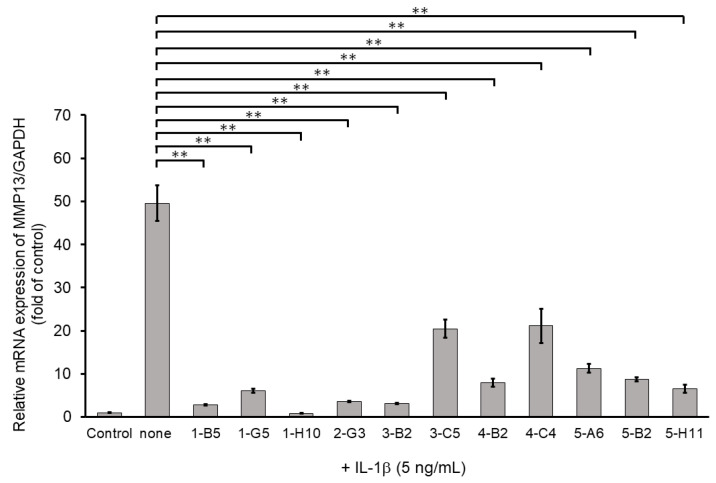
Inhibitory effects of candidate compounds on IL-1β-induced MMP13 mRNA expression. Cells were treated with each compounds (10 µg/mL) for 24 h, followed by the stimulation with IL-1β (5 ng/mL) for 6 h. MMP13 mRNA level was calculated relative to that in uninduced control cells. Values represent mean ± SE (*n* = 6 per group). ** *p* < 0.01.

Among ECM-degrading proteinases, MMP13 expression is induced during inflammation and plays a crucial role in OA development by degrading articular cartilage-specific ECM components, especially collagen type II (COL2A1) [10]. Therefore, we examined the inhibitory effects of 345 compounds on MMP13 mRNA expression using IL-1β-stimulated OUMS27. As shown in Figure 2, stimulation with IL-1β at 5 ng/mL significantly increased the mRNA expression of MMP13 compared to that in the unstimulated control. Among 345 molecules, 11 showed significant suppression of IL-1β-induced MMP13 expression as compared with IL-1β alone treatment group (Figure 2). Full name of each compounds was indicated in the Supplemental Table S1.

### 2.3. Screening Based on Inhibitory Effects on IL-1β-Induced ADAMTS4 and ADAMTS9 mRNA Expression

**Figure 3:**
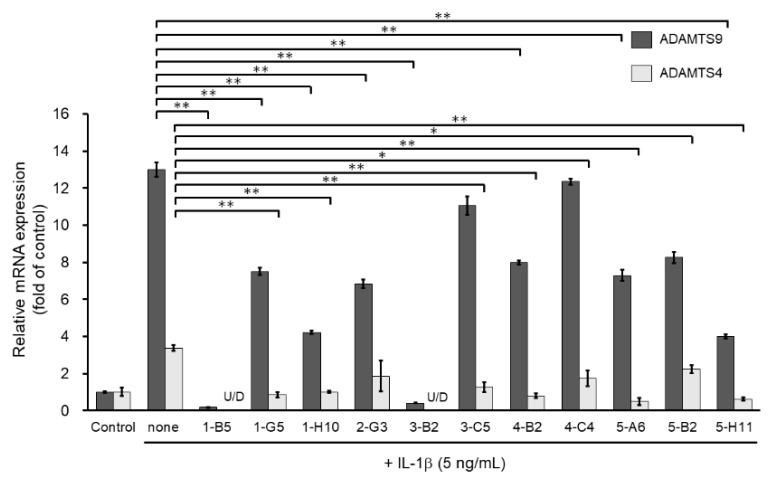
Inhibitory effects of candidate compounds on IL-1β-induced ADAMTS4 and ADAMTS9 mRNA expression. Cells were treated with each compounds (10 µg/mL) for 24 h, followed by the stimulation with IL-1β (5 ng/mL) for 6 h. ADAMTS4 and ADAMTS9 mRNA levels were calculated relative to their mRNA levels in uninduced control cells. Values represent mean ± SE (*n* = 6 per group). * *p* < 0.05 and ** *p* < 0.01, respectively.

We then screened the 11 MMP13-suppressing compounds by examining their effects on mRNA expression of ADAMTS4 and ADAMTS9. ADAMTS4 and ADAMTS9 are ECM-degrading enzymes that cleave cartilage-specific proteoglycans (aggrecan) and are induced by IL-1β stimulation. Among the 11 hit molecules, seven compounds significantly suppressed the mRNA expression of both IL-1β-induced ADAMTS4 and ADAMTS9 (** *p* < 0.01) (Figure 3). Among them, 1-B5 was a compound called berberine chloride, one of the benzylisoquinoline alkaloids derived from several species of medicinal herbs, such as *Berberis vulgaris*, *Berberis aristate*, and *Coptis chinensis*. Several previous studies have reported that this compound is an effective therapeutic agent for OA as it has multifunctional (multiple pharmacological) effects (various biological activities), including anti-inflammatory, anti-cancer, antioxidant (anti-oxidative), anti-apoptotic, and chondroprotective effects [11,12,13,14]. Therefore, berberine chloride was excluded from further screening. From the remaining six compounds, we selected three compounds, 1-H10, 3-B2, and 5-H11, which showed strong suppression effects (50% or higher) on IL-1β-induced gene expression of ADAMTS9 (Figure 3).

### 2.4. Screening via Inhibitory Effects on IL-1β-Induced MMP3 and COX-2 mRNA Expressions

**Figure 4:**
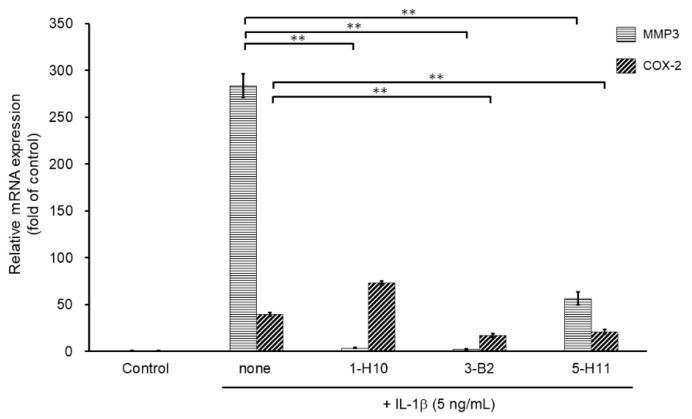
Inhibitory effects of candidate compounds on IL-1β-induced MMP3 and COX-2 mRNA expression. Cells were treated with each compounds (10 µg/mL) for 24 h, followed by the stimulation with IL-1β (5 ng/mL) for 6 h. Levels of MMP3 and COX-2 mRNAs levels were calculated relative to their mRNA levels in uninduced control cells. Values represent mean ± SE (*n* = 6 per group). ** *p* < 0.01.

To select more efficient candidate compounds, we further examined the three hit compounds by analyzing their inhibitory effects of MMP3 and COX-2 mRNA expression. MMP3 is an ECM-degrading enzyme produced by synovial cells and chondrocytes. It is stimulated by inflammatory cytokines and is involved in articular cartilage destruction by cleaving proteoglycans and collagen. As shown in Figure 4, MMP3 mRNA expression was significantly suppressed by all three compounds. However, compound 5-H11 showed a slightly smaller inhibitory effect on MMP3 expression than that of the other two compounds. COX-2 is an inflammatory mediator that plays an important role in various inflammatory diseases. COX-2 mediates the conversion of arachidonic acid to prostaglandin E_2_ (PGE_2_) and prostacyclin (PGI_2_). COX-2 is induced by stimulation with cytokines and growth factors. IL-1β stimulation significantly increased the mRNA expression level of COX-2, whereas compounds 3-B2 and 5-H11 significantly suppressed COX-2 expression. However, 1-H10 did not suppress COX-2 expression. Based on these results, compound 3-B2 was selected as the most promising candidate (Figure 4).

### 2.5. Effects of Compound 3-B2 on the Viability of IL-1β-Treated OUMS27 Cells

**Figure 5:**
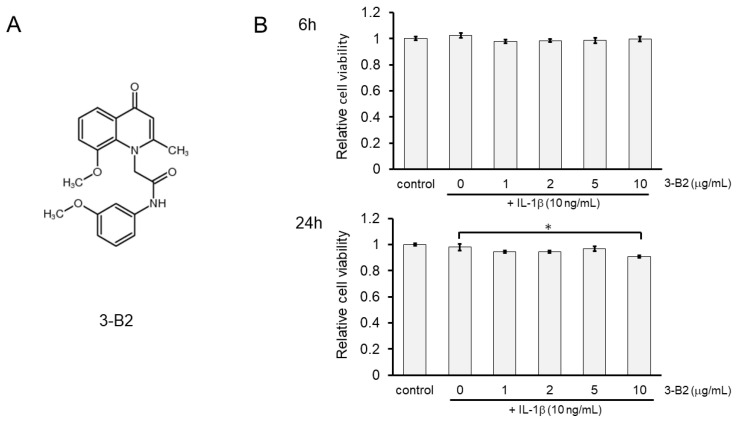
Effect of 3-B2 on the cell viability of OUMS-27 cells and IL-1β-induced damage. (**A**) Structure of the selected compound, 3-B2. (**B**) Relative cell viability determined by MTS assay shows pretreatment with 3-B2 at 1–10 μg/mL concentrations did not have significant cytotoxic effects on OUMS-27 cells after 6 h of IL-1β-stimulation, while 3-B2 pretreatment (24 h) at 10 μg/mL with longer IL-1β stimulation (24 h) has moderate cytotoxic effects on OUMS-27 cells. Values represent mean ± SE (*n* = 6 per group). * *p* < 0.05.

The chemical structure of 3-B2 is shown in Figure 5A. The effects of 3-B2 on the viability of IL-1β-induced OUMS27 cells were further analyzed using the MTS assay. Cells were pretreated with various concentrations of 3-B2 (0, 1, 2, 5, and 10 μg/mL) for 24 h, followed by stimulation with IL-1β (10 ng/mL) for 6 or 24 h. We found that 3-B2 pretreatment at concentrations of 1–10 μg/mL did not have cytotoxic effects after 6 h of IL-1β-stimulation (Figure 5B); however, pretreatment with this compound at 10 μg/mL was moderately cytotoxic in OUMS27 cells treated with IL-1β for 24 h. The results indicated that 3-B2 had no significant cytotoxicity against IL-1β-induced OUMS27 cells, except at 10 μg/mL for 24 h (* *p* < 0.05).

### 2.6. 3-B2 Inhibited IL-1β-Induced Gene Expression of Articular Cartilage ECM-Degrading Proteases in OUMS27 Cells

**Figure 6:**
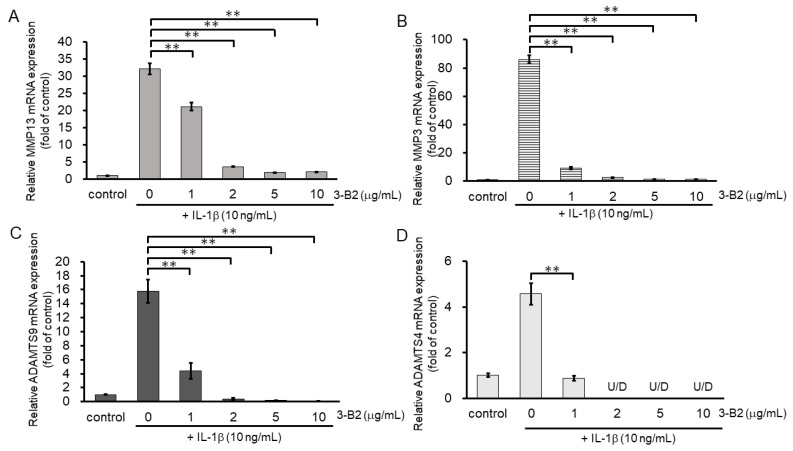
Inhibitory effects of 3-B2 on IL-1β-induced MMP and ADAMTS mRNA expression. OUMS-27 cells were pretreated with 3-B2 at various concentrations (0, 1, 2, 5, and 10 μg/mL) and then incubated with IL-1β (10 ng/mL) for 6 h (**A**) MMP-13, (**B**) MMP3, (**C**) ADAMTS9, (**D**) ADAMTS4. Values represent mean ± SE (*n* = 4 per group). ** *p* < 0.01.

We then investigated the inhibitory effect of 3-B2 on the mRNA expression of ECM-degrading enzymes including MMP13, MMP3, ADAMTS9, and ADAMTS4 in IL-1β-stimulated OUMS27 cells at various concentrations (0, 1, 2, 5, and 10 μg/mL) (Figure 6A–D). After 6 h of stimulation with 10 ng/mL of IL-1β, the mRNA expression of these ECM-degrading enzymes was upregulated, whereas 3-B2 significantly inhibited this mRNA expression in a concentration-dependent manner (Figure 6A–D).

### 2.7. Inhibitory Effects of 3-B2 on the Protein Expression of Articular Cartilage ECM-Degrading Proteases in OUMS27 Cells

**Figure 7:**
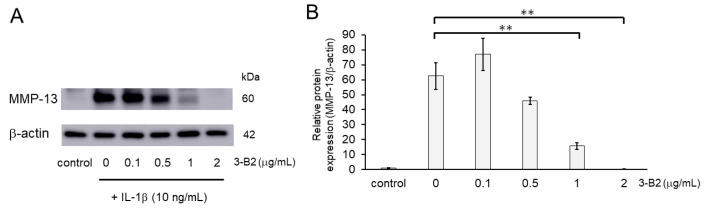
Inhibitory effects of 3-B2 on IL-1β-induced MMP-13 protein expression. (**A**) Western blot analysis of the relative protein level of MMP-13. Pre-treatment with 3-B2 for 6h significantly decreased the protein expression of MMP-13, which was induced by IL-1β (10 ng/mL) in a dose-dependent manner. (**B**) Quantitative estimation of the protein levels of MMP-13 after 3-B2 pre-treatment and IL-1β induction. Values represent mean ± SE (*n* = 4 per group). ** *p* < 0.01.

We further evaluated the effect of 3-B2 on the protein expression levels of MMP-13 in IL-1β-induced OUMS27 using western blot analysis. Cells were pretreated with different concentrations of 3-B2 (0, 0.1, 0.5, 1, or 2 μg/mL for 24 h), followed by stimulation with or without IL-1β (10 ng/mL) for 24 h. As shown in Figure 7, 3-B2 pre-treatment significantly decreased the protein expression of MMP-13, which was induced by IL-1β in a dose-dependent manner.

### 2.8. 3-B2 Inhibited IL-1β-Induced MAPK Activation in OUMS27 Cells

**Figure 8:**
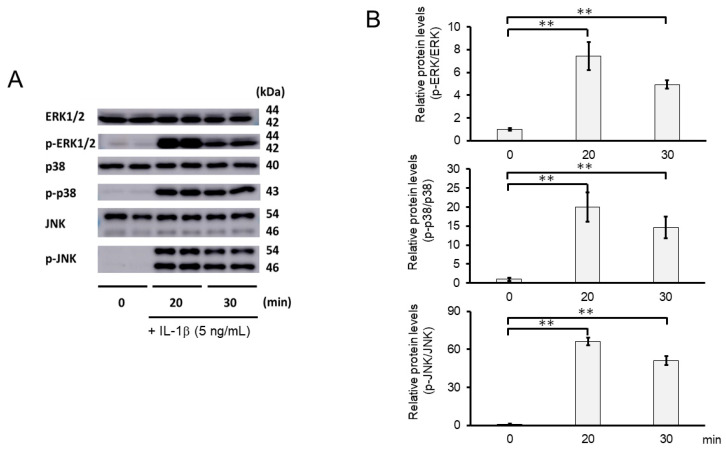
Activation of MAPK signaling pathway in IL-1β-stimulated OUMS27 cells. (**A**) Western blot analysis of the relative phosphorylation levels of Erk1/2, p38, and JNK after IL-1β treatment. Phosphorylation of Erk1/2, p38, and JNK increased significantly after 20 min of treatment with 5 ng/mL IL-1β, and decreased after 30 min. (**B**) Quantitative estimation of the relative phosphorylated protein levels of Erk1/2, p38, and JNK at given time points. Values represent mean ± SE (*n* = 3 per group). ** *p* < 0.01.

**Figure 9:**
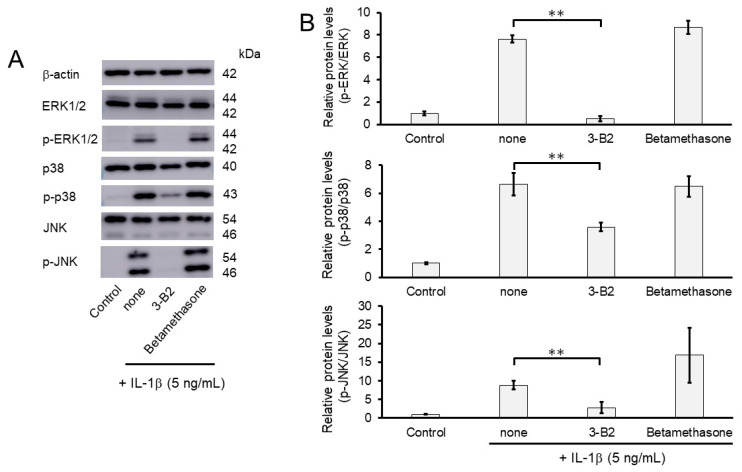
Effect of 3-B2 and betamethasone on the phosphorylation of Erk1/2, p38, and JNK in IL-1β-stimulated OUMS27 cells. (**A**) Western blot analysis of the relative phosphorylation levels of Erk1/2, p38, and JNK after 3-B2 or betamethasone treatment. Cells were treated with 10 ng/mL of 3-B2 or betamethasone for 24 h and then induced with 5 ng/mL IL-1β for 20 min. Treatment with 3-B2 but not betamethasone significantly reduced the phosphorylation of Erk1/2, p38, and JNK. (**B**) Quantitative estimation of the relative phosphorylated protein levels of Erk1/2, p38, and JNK after 3-B2 or betamethasone treatment. Values represent mean ± SE (*n* = 3 per group). ** *p* < 0.01.

**Figure 10:**
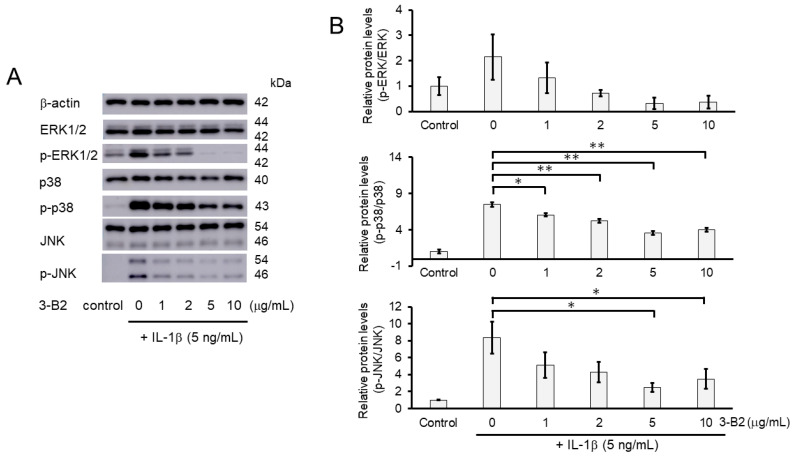
Activation of the MAPK signaling pathway was suppressed by 3-B2 in a concentration-dependent manner. (**A**) Western blot analysis of the relative phosphorylation levels of Erk1/2, p38, and JNK after 3-B2 and IL-1β treatment. Cells were treated with 3-B2 at various concentrations (0, 1, 2, 5, and 10 μg/mL) and then induced with 5 ng/mL IL-1β for 20 min. 3-B2 reduced the phosphorylation of Erk1/2, p38, and JNK in a dose-dependent manner. (**B**) Quantitative estimation of the relative phosphorylated protein levels of Erk1/2, p38, and JNK after 3-B2 and IL-1β treatment. Values represent mean ± SE (*n* = 3 per group). * *p* < 0.05 and ** *p* < 0.01, respectively.

We further investigated whether the inhibitory mechanisms of 3-B2 on the overexpression of ECM-degrading enzymes such as MMP13, MMP3, ADAMTS4, and ADAMTS9 was related to the MAPK signaling pathway in IL-1β-induced OUMS27. MAPK signaling is known to be activated by IL-1β stimulation, and to mediate inflammatory responses, and plays an important role in OA pathogenic processes, such as cartilage degradation. First, we examined the kinetics of phosphorylation in MAPK signaling molecules including Erk1/2, p38, and JNK in IL-1β-stimulated OUMS27 cells (0, 20, and 30 min). As shown in Figure 8A,B, the phosphorylation levels of Erk1/2, p38, and JNK were significantly upregulated, peaked after treatment with 5 ng/mL IL-1β for 20 min, and then decreased. We thus confirmed that the phosphorylation of each signaling molecule was transiently activated by IL-1β under the same experimental condition. We next evaluated whether 3-B2 suppresses the IL-1β-upregulated phosphorylation of Erk1/2, p38, and JNK in OUMS27 cells. Cells were treated with 10 ng/mL of 3-B2 or betamethasone, a steroid drug, for 24 h and then induced with 5 ng/mL IL-1β for 20 min. Betamethasone is a corticosteroid hormone used as an anti-inflammatory and immunosuppressive drug; intra-articular injections of betamethasone have been clinically administered for OA treatment. Betamethasone has potent glucocorticoid activity and inhibits the overexpression of ECM-degrading enzymes, such as MMP13 and ADAMTS4. Betamethasone was used as a negative control in this study because it suppresses the expression of ECM degrading enzymes, such as MMP13, via another pathway without suppressing MMPK signaling. The results showed that 3-B2 significantly reduced the phosphorylation of Erk1/2, p38, and JNK; however, betamethasone did not inhibit their phosphorylation (Figure 9A,B). Furthermore, 3-B2 significantly suppressed the activation of the MAPK signaling pathway molecules (phosphorylation levels) in a concentration-dependent manner (Figure 10A,B).

### 2.9. 3-B2 Synergistically Attenuated MMP13 and ADAMTS9 Expression with Betamethasone in IL-1β-Stimulated OUMS-27 Cells

**Figure 11:**
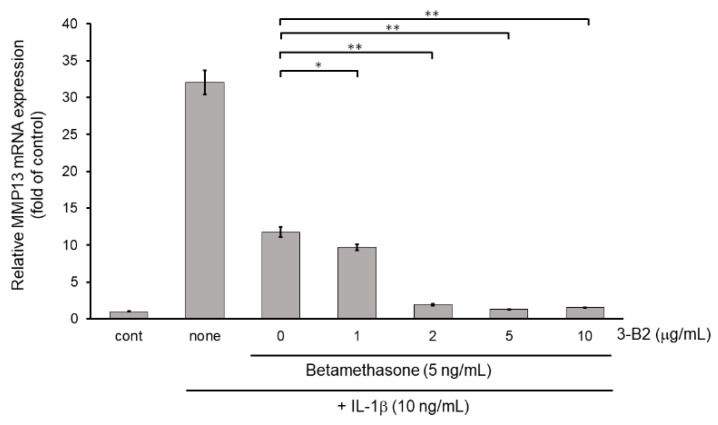
Additive effect of 3-B2 on attenuating MMP13 mRNA expression with low dose of betamethasone in IL-1β-treated OUMS-27 cells. Cells were pretreated with various concentrations (1, 2, 5, 10 μg/mL) of 3-B2 and with betamethasone (5 ng/mL), followed by the stimulation with IL-1β (5 ng/mL) for 6 h. Along with low dose of betamethasone, 3-B2 exhibited significant additive attenuating effect on MMP13 mRNA expression in IL-1β-stimulated OUMS-27 cells. Values represent mean ± SE (*n* = 6 per group). * *p* < 0.05 and ** *p* < 0.01, respectively.

**Figure 12:**
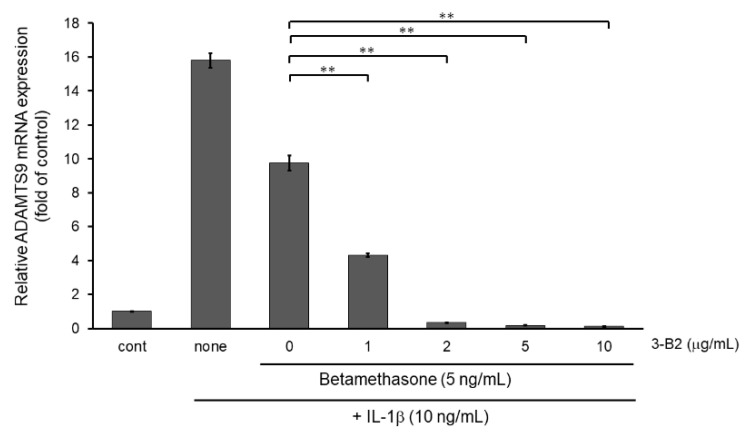
Additive effect of 3-B2 on attenuating ADAMTS9 mRNA expression with low dose of betamethasone in IL-1β-stimulated OUMS-27 cells. Cells pretreated with various concentrations (1, 2, 5, 10 μg/mL) of 3-B2 and betamethasone (5 ng/mL), followed by the stimulation with IL-1β (5 ng/mL) for 6 h. Along with low dose of betamethasone, 3-B2 exhibited significant additive attenuating effect on ADAMTS9 mRNA expression in IL-1β-stimulated OUMS-27 cells. Values represent mean ± SE (*n* = 6 per group). ** *p* < 0.01.

Next, we examined whether the combination of 3-B2 and betamethasone could synergistically attenuate MMP13 and ADAMTS9 expression. As shown in Figure 11, 3-B2 had an additive effect on attenuating MMP13 in combination with a low dose (5 ng/mL) of betamethasone in a dose-dependent manner in IL-1β stimulated OUMS-27 cells. Further, 3-B2 effectively attenuated ADAMTS9 expression in combination with a low dose (5 ng/mL) of betamethasone in a dose-dependent manner (Figure 12).

## 3. Discussion

MMPs play a major role in the development of OA. In this study, we screened a chemical library provided by RIKEN to explore novel promising therapeutic agents (chemical compounds) that could suppress IL-1β-induced upregulation of matrix metalloproteinases such as MMP13. We identified a novel and promising candidate compound, 3-B2, and further clarified its underlying molecular mechanism. We found that 3-B2 significantly suppressed the mRNA and protein expression of MMP13. Further, 3-B2 significantly suppressed the mRNA expression of MMP3, ADAMTS4, ADAMTS9 and COX-2. This additive effect is favorable because MMP3, ADAMTS4, and ADAMTS9 are thought to be involved in OA progression. Furthermore, as COX-2 is an enzyme that promotes inflammation, suppression of COX-2 expression may further suppress excessive inflammatory responses. Therefore, attenuation of COX-2 by 3-B2 can provide more advantages. Based on these effects, 3-B2 may be a promising candidate for treating OA. We also confirmed that this compound significantly suppressed the activation of MAPK signaling pathway molecules such as Erk1/2, p38, and JNK in IL-1β-induced OUMS27 cells in a concentration-dependent manner. MAPK signaling is one of the key signaling pathways that mediate inflammation responses and cartilage degradation and promote the pathological progression of OA [9,15,16]. These results indicate that 3-B2 could attenuate MMP and ADAMTS expression as well as chondrocyte inflammation by suppressing MAPK signaling pathway activation. Collectively, our results suggest that 3-B2 may be a promising therapeutic agent for OA.

3-B2 (2-(8-methoxy-2-methyl-4-oxoquinolin-1(4H)-yl)-N-(3-methoxyphenyl) acetamide) is synthesized from tryptophan metabolites. Our data indicate that 3-B2 can attenuate the phosphorylation of ERK and p38. Further, 3-B2 can strongly attenuate JNK phosphorylation These pathways play a central role in IL-1β-induced MMP13 expression. Based on the information in the Pubchem database of NCBI, 3-B2 has interesting potential properties [17]. 3-B2 can inhibit transcriptional enhanced associate domain (TEAD)-Yes-associated protein (YAP) interactions. YAP activation is also involved in OA progression [18,19,20]. Although we did not examine the YAP pathway in this study, 3-B2 may have diverse functions that are favorable for protecting cartilage; these need to be analyzed in a future study.

This study had several limitations. Firstly, we used OUMS-27, a chondrosarcoma cell line, rather than chondrocytes from patients with OA. Previous studies showed that OUMS-27 cells express proteoglycans such as aggrecan as well as collagen types I, II, III, IX, and XI, which stably maintain the properties of differentiated chondrocytes [21,22]. We previously reported that OUMS-27 cells produced ADAMTS mRNA following IL-1β stimulation. In the current study, MMP13 was also increased due to IL-1β stimulation in OUMS-27 cells at the RNA and protein levels. We also confirmed that anabolic factors such as type II collagen and aggrecan mRNA levels were decreased by IL-1β stimulation. IL-1β stimulation of OUMS-27 cells resulted in unbalanced gene expression between anabolic factors and catabolic factors. We also clarified that the intracellular signaling pathway activation in IL-1β-stimulated OUMS27 cells was similar to that in IL-1β-stimulated OA chondrocytes. Therefore, we applied this chondrocytic property of OUMS-27 cells to examine the gene expression levels of MMPs and ADAMTSs in them using chemical compound library screening. In fact, several hit chemical compounds were previously reported by another group as potential novel OA drugs in vitro (i.e., using OA chondrocytes) and in vivo. These data also support that our screening system using OUMS-27 cells was valid.

We screened a pilot library focusing on metalloproteinase expression. rather than protease activity. Various strategies can be used to evaluate anti-OA drug candidates. At present, NSAIDS are the most widely used anti-OA drugs. Selective COX-2 inhibitors are also promising as anti-OA drugs. However, cardiovascular side effects related to COX-2 inhibitors have been reported. Corticosteroids, including betamethasone, are strong anti-inflammatory drugs for controlling pain, swelling, and MMP/ADAMTS expression. Corticosteroids are typically locally administered, including through intra-articular injection. However, overdose or long use of corticosteroids induces notable side effects such as diabetes mellitus, atherosclerosis, and osteoporosis. Therefore, decreasing the dosage of corticosteroids by using supportive chemical compounds may benefit patients with OA; this should be evaluated in further studies. In this study, we found that the combination of 3-B2 with betamethasone can achieve a small dose of betamethasone compared with that of betamethasone used alone. Considering the unacceptable side effects of high-dose and long-term betamethasone treatment, reducing the use of betamethasone through combination therapy with another drug is a potentially beneficial strategy, with the possibility of using 3-B2 as an OA drug candidate. However, the detailed combination conditions required for effective cartilage protection in early OA stage need to be examined in further studies. Our current data support that low-dose betamethasone with 3-B2 can be used as an adjunctive therapy, which we will further analyze in an early-stage OA model in vivo. A promising strategy may be introducing metalloproteinase inhibitors that can block ECM degradation by MMPs/ADAMTSs. MMPs are involved in various diseases, including OA, cardiovascular diseases, neurodegenerative diseases, and cancers. Some compounds, such as marimastat, the carboxylate BAY12-9566, protect against matrix degradation by inhibiting some MMPs. However, clinical trials using broad-spectrum MMP inhibitors have been restricted because patients tend to develop musculoskeletal syndromes such as arthralgia and myalgia because of the high structural similarities among members of the MMP family. It may be possible to avoid unfavorable side effects by using candidates with mild MMP inhibitory effects. Therefore, we first screened a chemical compound library in an MTS assay. We used the attenuation of IL-1β-induced MMP13 mRNA expression in OUMS27 cells as the second major evaluation criterion. This strategy revealed several positive candidates, including known anti-OA molecules, as hits. Betamethasone was the most effective compound in this library and is widely recognized as an anti-OA drug. Several other compounds have also been reported to be effective against OA. For example, berberine is reported to exert protective effects in a rat OA model by attenuating MMP-3 [23]. Further studies have supported this finding, demonstrating that berberine has anti-catabolic and anti-inflammatory abilities through MAPKs in IL-1β-induced rat chondrocytes [24]. These results indicate that our strategy was valid.

At last, this study was conducted in vitro. The novel compound, 3-B2, has not yet been examined in vivo. As a potential drug for OA, it is essential to examine its effects in an in vivo model of OA [25]. However, considering the limited safety range of 3-B2 based on its cytotoxic effects, we consider that 3-B2 can be used as an anti-OA drug at a low dose. From this viewpoint, searching an alternative compound to 3-B2, with a wider safety range is necessary for animal studies as a novel therapeutic candidate.

## 4. Materials and Methods

### 4.1. Reagents

A library of chemical compounds (NPDepo Pilot library) at 10 mg/mL in 100% dimethyl sulfoxide (DMSO) was obtained from RIKEN (Wako, Saitama, Japan). The chemical arrays were prepared using a previously described method [26]. The library comprised 400 small-molecule compounds, including natural products or synthetic natural product derivatives. Recombinant human IL-1β was purchased from R&D Systems (Minneapolis, MN, USA), stored at −80 °C, and diluted in culture medium immediately before use. Betamethasone powder was obtained from Tokyo Chemical Industry Co., Ltd. (Tokyo, Japan), and 3-B2 (2-(8-methoxy-2-methyl-4-oxoquinolin-1(4H)-yl)-N-(3-methoxyphenyl)acetamide) powder was obtained from Namiki Shoji Co., Ltd. (Tokyo, Japan); both compounds were dissolved in 100% DMSO, and stored at −30 °C until use. Anti-MMP13 (F-89) antibody was obtained from Kyowa Pharma Chemical Co., Ltd. (Toyama, Japan). Anti-p44/42 MAPK (Erk1/2) (#4695), anti-phospho-p44/42 MAPK (Erk1/2) (#4370), anti-p38 MAPK (#9212), anti-phospho-p38 MAPK (#4511), anti-SAPK/JNK (#9252), and anti-phospho-SAPK/JNK (#4668) antibodies were purchased from Cell Signaling Technology, Inc. (Danvers, MA, USA). Anti-β-actin antibody (A5441) was obtained from Sigma-Aldrich (St. Louis, MO, USA). The secondary antibodies used were HRP-conjugated goat anti-mouse IgG (Sigma Aldrich or R&D Systems), and HRP-conjugated goat anti-rabbit IgG (SeraCare Life Sciences, Inc., Milford, MA, USA) [9,27].

### 4.2. Cell Culture and Treatment

OUMS-27 chondrosarcoma cells were prepared as previously described [9,28]. Cells were cultured in Dulbecco’s modified Eagle’s medium (DMEM, Sigma-Aldrich, St. Louis, MO, USA) supplemented with 10% fetal bovine serum, 100 U/mL penicillin, and 100 U/mL streptomycin at 37 °C under 5% CO_2_ and 20% O_2_ in a humidified chamber. OUMS-27 cells were seeded at 1.0 × 10^5^ cells/well in 12-well tissue culture plates and cultured for 2 days. After changing the medium or serum-free medium and incubating for another 24 h, the cells were pretreated with each test compound in the library (10 μg/mL) for 24 h, and then exposed to IL-1β (either 5 ng/mL or 10 ng/mL) for 6 h /24 h as indicated in each experiment. The chemical compounds were dissolved in DMSO. The final concentration of DMSO in assays using OUMS-27 cells was less than 1.0% (*v*/*v*) and did not affect cell viability.

### 4.3. Cell Viability Assay

As an initial screening, the effects of 400 chemical compounds from the library on the viability of OUMS-27 cells were investigated using a CellTiter 96 AQueous One Solution Cell Proliferation Assay (MTS) kit (Promega, Madison, WI, USA) according to the manufacturer’s instructions, as previously described [29,30]. The optical density (OD) was measured at a wavelength of 490 nm. Cells from passages 7–12 were used for all experiments. Cells were seeded at 5.0 × 10^3^ cells/well in 96-well tissue culture plates and cultured for two days. After incubation, the medium was replaced with fresh medium and the cells were incubated overnight. The medium was then changed to culture medium containing 10 μg/mL of each test compound in the library and incubated for 24 h. Cell viability was expressed as the percentage of proliferation compared with that of control cells treated with only 1.0% DMSO. After determination of 3-B2 as a promising candidate compound, cytotoxicity in OUMS-27 was evaluated by treatment with various concentrations (1, 2, 5, and 10 μg/mL) of 3-B2 for 24 h, followed by stimulation with IL-1β (5 or 10 ng/mL) for 6 h or 24 h at 37 °C, using an MTS assay kit as previously described.

### 4.4. RNA Extraction and Quantitative Real-Time (qRT) -PCR

Following cytokine stimulation, total RNA was extracted using TRIzol reagent (Invitrogen, Carlsbad, CA, USA) according to the manufacturer’s instructions and was quantified by measuring the absorbance at 260 nm using an Eppendorf BioPhotometer D30 (Eppendorf, Hamburg, Germany) as previously described [31,32,33]. The extracted total RNA (2 µg) was subjected to reverse transcription using ReverTra Ace (Toyobo, Osaka, Japan) at 30 °C for 10 min, 42 °C for 60 min, and 85 °C for 10 min (99 °C for 5 min) with random primers (Toyobo). Quantitative real-time PCR was performed on a StepOnePlus system (Applied Biosystems, Foster City, CA, USA) using Taqman Fast Advanced Master Mix (Thermo Fisher Scientific, Waltham, MA, USA), as previously reported [34,35]. The reaction mixture contained 5 μL of 2× TaqMan Fast Advanced Master Mix, 0.5 μL of TaqMan Gene Expression assays for target genes (ADAMTS4, ADAMTS9, MMP3, MMP13, or COX-2) and the endogenous internal control gene (glyceraldehyde 3-phosphate dehydrogenase [GAPDH]), along with 4 μL cDNA. The reaction conditions were as follows: 95 °C for 20 s, followed by 40 cycles of 95 °C for 1 s and 60 °C for 20 s. TaqMan primers and probes used for the analysis were as follows: human ADAMTS4, Hs00192708_m1; ADAMTS9, Hs00172025_m1; MMP3, Hs00968305_m1; MMP13, Hs00233992_m1; COX-2, Hs00153133_m1; GAPDH, Hs99999905_m1 (Applied Biosystems, Foster City, CA, USA). The mRNA expression levels of target genes was normalized to GAPDH using the comparative Ct ^(ΔΔCT)^ method as previously described [36,37,38].

### 4.5. Western Blot Analysis

OUMS-27 cells were plated at a density of 1 × 10^5^ cells/well in 6-well plates, and cultured for 3 days. After compound pretreatment and IL-1β stimulation, the cells were washed once in cold Tris buffer saline (TBS) and lysed with ice-cold lysis buffer (20 mM Tris-HCL (pH 8.0), 150 mM NaCl, 0.5% Nonidet P-40, 0.5% Triton X-100, 1.0 mM EDTA, 0.5% sodium deoxycholate, 10% glycerol, and 10 mM NaF, 0.1% SDS) supplemented with Complete Mini protease inhibitor cocktail tablets (Roche Applied Science, Mannheim, Germany) or protease inhibitor cocktail (Nacalai, Kyoto, Japan) and phosphatase inhibitor cocktail 3 (Sigma-Aldrich). Cell lysates were centrifuged at 20,000× *g* for 15 min, and total protein concentrations were quantified using a Bio-Rad DC^TM^ Protein Assay Kit (Bio-Rad Laboratories, Hercules, CA, USA), with bovine serum albumin used as the standard protein. Cell lysates were mixed with 4× reducing sample buffer, denatured at 95 °C for 5 min, separated on 10% SDS-PAGE gels, and transferred onto polyvinylidene difluoride (PVDF) membranes (Merck Millipore Ltd., Darmstadt, Germany) as previously described [15,39]. The primary antibodies were anti-MMP13 (1:400), anti-ERK (1:1000), anti-phospho-ERK (1:1000), anti-p38 (1:1000), anti-phospho-p38 (1:1000), anti-JNK (1:1000), anti-phospho-JNK (1:1000), and anti-β-actin (1:10,000). Secondary antibodies for anti-mouse IgG (1:1000) and anti-rabbit IgG (1:5000) were used. After blocking with 5% skim milk in TBS-0.05% Tween 20 (TBST) for 1 h at room temperature, the membranes were incubated for 1 h at room temperature or overnight at 4 °C with the primary antibodies, followed by subsequent incubation with their respective HRP- conjugated secondary antibodies for 1 h at room temperature. Immunoreactive protein bands were visualized using the Amersham ECL prime kit (GE Healthcare, Buckinghamshire, UK) and analyzed using an Amersham Imager 600 (GE Healthcare) as previously described [15].

### 4.6. Statistical Analysis

All experiments were performed independently in triplicates. Results are presented as mean ± standard error of the mean (SE). Statistical analyses were performed using Student’s *t*-test for unpaired parametric correlations. Statistical significance was set at *p* < 0.05.

## Data Availability

The data presented in this study are available on request from the corresponding author.

## Supplementary Materials

Supplementary materials can be found at https://www.mdpi.com/article/10.3390/ijms23052681/s1.
