# Supplementary material for: Potential of a Novel Chemical Compound Targeting Matrix Metalloprotease-13 for Early Osteoarthritis: An In Vitro Study
Source: Int J Mol Sci. 2022 Feb 28;23(5):2681. doi: 10.3390/ijms23052681 (PMC8910651; doi:10.3390/ijms23052681)
Supplement: Supplementary file 1 [file ijms-23-02681-s001.zip › ijms-1596043-supplementary.pdf]

Supplementary Figure S1

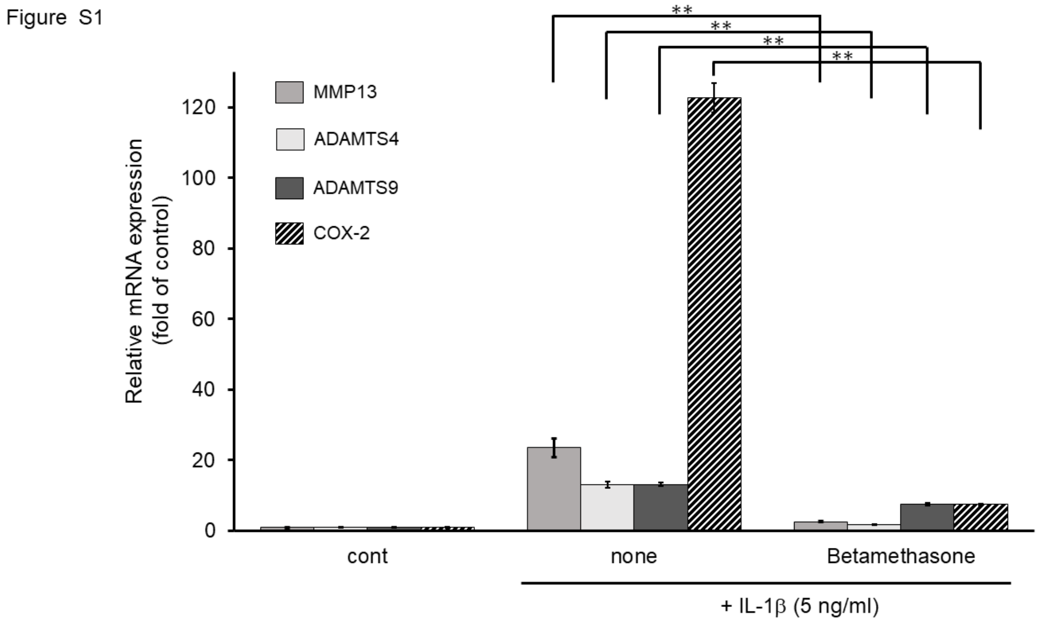

Figure S1. Inhibitory effect of betamethasone (100 μg/mL) on the expressions of various genes induced by IL-1βin OUMS-27 cells. MMP13, ADAMTS4, ADAMTS9, and COX2 were induced by IL-1β stimulation, and betamethasone attenuated these inductions. ** $p < 0.01$.

Supplementary Figure S2

Figure S2

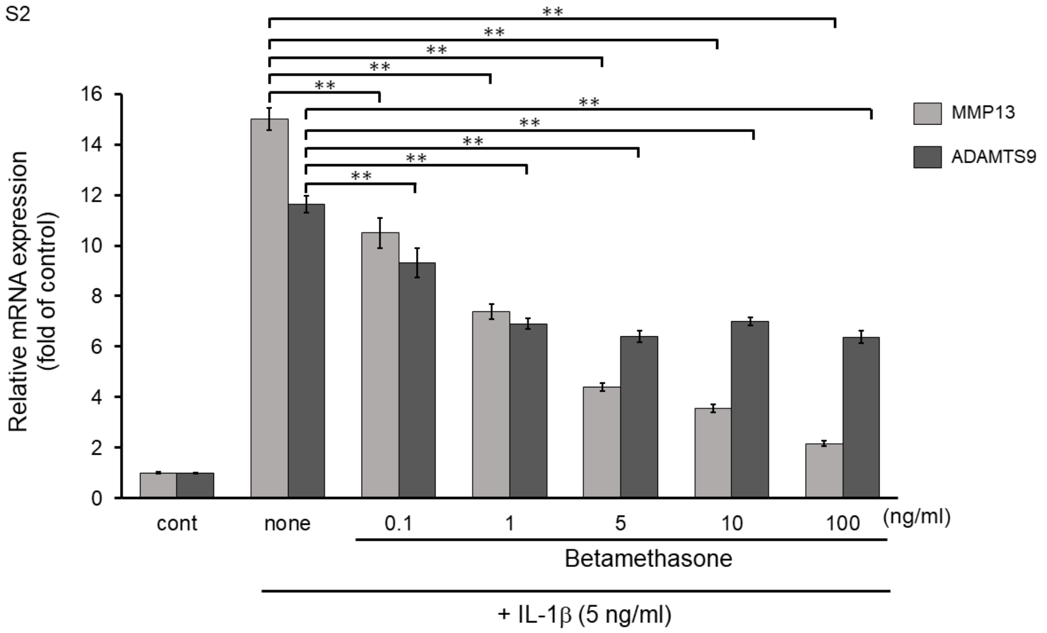

Figure S2. Dose-dependent inhibitory effect of betamethasone on the relative expression of MMP13 and ADAMTS9 mRNA in IL-1β-stimulated OUMS-27 cells. Cells were treated with 0.1, 1, 5, 10, and 100 ng/mL of betamethasone. Betamethasone exhibited inhibitory effect in a dose-dependent manner. ** $p < 0.01$

Table S1. The selected 11 compounds.

Selected Compound	Name
1-B5	Berberine chloride
1-G5	[2-[(10R,13S,17R)-11,17-dihydroxy-10,13-dimethyl-3-oxo-7,8,9,11,12,14,15,16-octahydro-6H-cyclopenta[a]phenanthren-17-yl]-2-oxoethyl] propanoate
1-H10	1-(2,3-dimethylphenyl)-6-hydroxy-5-(2,3,4,9-tetrahydro-1H-pyrido[3,4-b]indol-1-yl)pyrimidine-2,4-dione
2-G03	N-[2-(5-methoxy-1H-indol-3-yl)ethyl]-4-methyl-2-(3,4,5-trimethoxyphenyl)-1,3-thiazole-5-carboxamide
3-B2	2-(8-methoxy-2-methyl-4-oxoquinolin-1-yl)-N-(3-methoxyphenyl)acetamide
3-C5	(4Z)-4-[(1-methylindol-3-yl)methylidene]-2-phenyl-1,3-oxazol-5-one
4-B2	3-(1,3-benzothiazol-2-yl)-7-hydroxy-8-methyl-4-oxochromene-2-carboxylic acid
4-C4	3-ethyl-7-[2-(4-methoxyphenyl)-2-oxoethoxy]-4-methylchromen-2-one
5-A6	4-[[[4-[2-[(10R,13S,17R)-11,17-dihydroxy-10,13-dimethyl-3-oxo-2,6,7,8,9,11,12,14,15,16-decahydro-1H-cyclopenta[a]phenanthren-17-yl]-2-oxoethoxy]-4-oxobutanoyl]amino]methyl]cyclohexane-1-carboxylic acid
5-B2	(2Z)-7-[[bis(2-methoxyethyl)azaniumyl]methyl]-3-oxo-2-[(3,4,5-trimethoxyphenyl)methylidene]-1-benzofuran-6-olate
5H11	2-[2-[carboxymethyl-[(2-hydroxyphenyl)methyl]amino]ethyl-[(2-hydroxyphenyl)methyl]amino]acetic acid;hydrochloride
